# A Single-Center Study of Patients With Synchronous Primary Malignancy: A Case Series

**DOI:** 10.7759/cureus.32839

**Published:** 2022-12-22

**Authors:** Abeer I Alsulaimani, Layla M Alkhaldi, Sheikha A AlTawairqi, Arif Khurshid, Hamma A Abdulaziz, Abdulrahman G Alotaibi, Haifa O Alotaibi

**Affiliations:** 1 Medicine, Taif University, Taif, SAU; 2 General Surgery, Alhada Military Hospital, Taif, SAU; 3 Surgical Oncology, Mcgill University, Montreal, CAN; 4 Surgery, Taif University, Taif, SAU

**Keywords:** oncosurgery, neoplasms, multiple, malignancy, synchronous

## Abstract

Multiple primary malignant tumors (MPMT) can be defined as more than two different tumors synchronously or metachronously forming in the same organ or different organs. The incidence of MPMTs varies dramatically between antemortem and postmortem examinations, becoming a serious medical issue. Evidence shows that the overall incidence of MPMTs is between 2.4% and 17%. Double primary malignancy (DPM) is considered the most common type of MPMT. In this case series, we present three cases of MPMT. The first case involved the colon and the breast, the second case involved the colon and the kidney, and the third case involved rectum and kidney.

## Introduction

Multiple primary malignant tumors (MPMT) can be defined as more than two different tumors synchronously or metachronously forming in the same organ or different organs. Coincidently with the increasing interest in cancer and with advances in both radical treatments of malignant tumors and diagnosis techniques, the trend has been toward increasing frequency of multiple malignant tumors [1]. Cancer survivors have a 20% higher risk of developing a new primary cancer than in general population [2]. A double primary malignancy (DPM) is defined as a second primary malignancy (SPM) that occurs in another organ(s) apart from the first primary malignancy [3]. DPM is considered the most common type of MPMT [2]. Up to 10% of cancer patients have been reported to develop DPM at different organ sites during a 10-year period after the surgical removal of the first primary tumor. DPM may be diagnosed synchronously (the second primary cancer is diagnosed within six months after the detection of the first primary cancer) or metachronously (the second primary cancer is diagnosed more than six months after the detection of the first primary cancer) [4]. In one study, that studied 145 patients with DPM, a synchronous second primary malignancy occurred in 57 patients (39.3%), and nearly half of the metachronous second primary malignancies (88 patients, 60.7%) occurred less than three years after the initial operation for the primary colorectal cancer [3]. We report three cases of double primary malignancy. The first case involved the colon and the breast, the second case involved the colon and the kidney, and the third case involved rectum and kidney.

## Case presentation

Case 1

**Figure 1:**
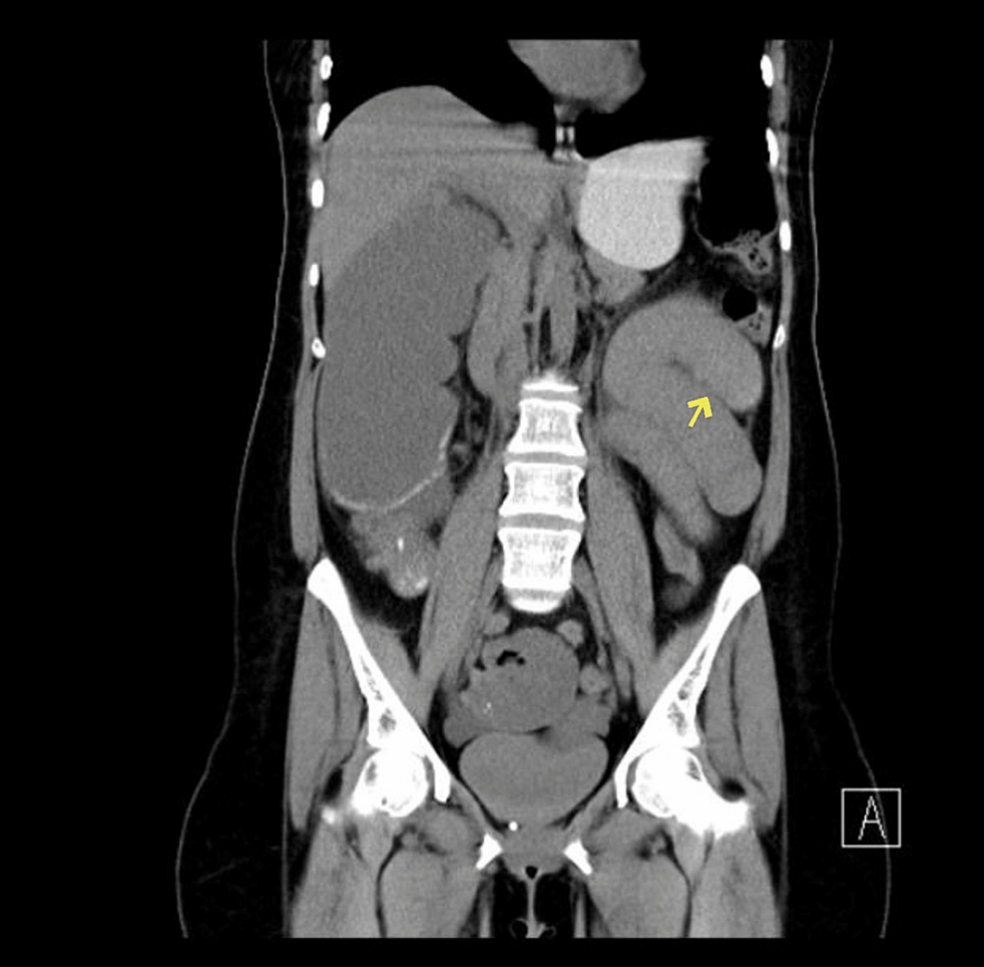
Case 1 - pre- and post-enhanced CT abdomen with oral and rectal contrast. It demonstrated a complete small and large bowel obstruction. At the level of the splenic flexure, there was small annular constricting thickening with transition zone measuring 3.2 cm.

In March 2020, a 46-year-old female with no significant past medical history presented to the emergency department (ED) with 10 days history of constipation and eight days history of vomiting. The patient was admitted as a case of intestinal obstruction. Her family history was positive for breast cancer; however, no genetic testing was done. A thorough physical examination revealed a distended and tender abdomen with an empty rectum. The blood work showed a low level of hemoglobin (9.5 g/dL), low mean corpuscular volume (MCV) (61.1 fL), and low mean corpuscular hemoglobin (MCH) (17.4 pg) that indicate microcytic hypochromic anemia. The tumor markers alpha-fetoprotein (AFP) and carcinoembryonic antigen (CEA) were within the normal levels. Pre- and post-enhanced CT abdomen with oral and rectal contrast was performed, which demonstrated a complete small and large bowel obstruction (Figure 1).

**Figure 2:**
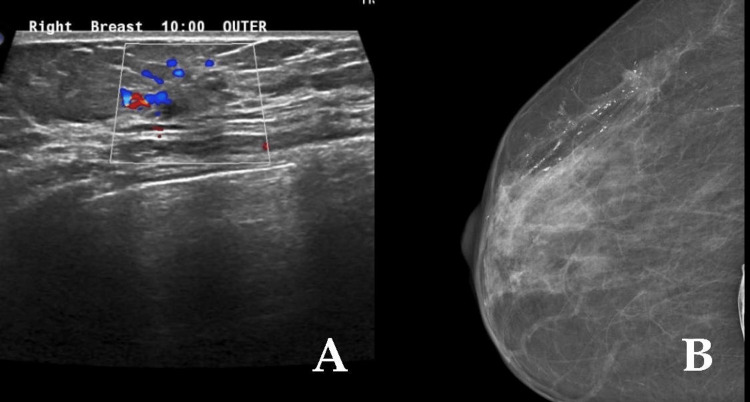
Case 1 - ultrasound and mammography. (A) Right breast US and (B) right breast mammography revealed that the right breast was BIRADS 4C. BIRADS: breast imaging-reporting and data system

At the level of the splenic flexure, there was small annular constricting thickening with transition zone measuring 3.2 cm. The patient underwent open extended left hemicolectomy with primary colo-colonic anastomosis. Histopathological examination of the resected colon showed a 3 cm mass in the splenic flexure, moderately differentiated adenocarcinoma infiltrating through the muscularis propria into the tissues surrounding the colon (PT3), 14 regional lymph nodes were free of the tumor (PN0). The margins were free from malignancy. The staging CT was negative for distant metastasis (M0). The patient had received four cycles of adjuvant chemotherapy and, during the last session, breast masses on both breasts were discovered. Bilateral breast ultrasound (US) and mammography were done to evaluate the lesions and revealed that the left breast was breast imaging-reporting and data system (BIRADS) 4A and right breast was BIRADS 4C (Figures 2A, 2B). The examination of right breast biopsy was suspected of poorly differentiated mammary carcinoma and the left breast biopsy was inconclusive. The patient underwent bilateral mastectomy and bilateral sentinel lymph node biopsies of both axillae.

The histopathological analysis of the bilateral breast specimens revealed right invasive ductal carcinoma, tumor size was 1.9 cm, (grade 3) with Paget’s disease of the nipple and fibroadenoma in the left breast. The tumor was positive for estrogen, progesterone receptors, and human epidermal growth factor receptor 2 (HER-2 neu). Left and right axilla, sentinel lymph nodes were negative for metastasis. Post-operatively the patient received adjuvant chemotherapy, radiotherapy, and hormonal therapy.

Case 2

**Figure 3:**
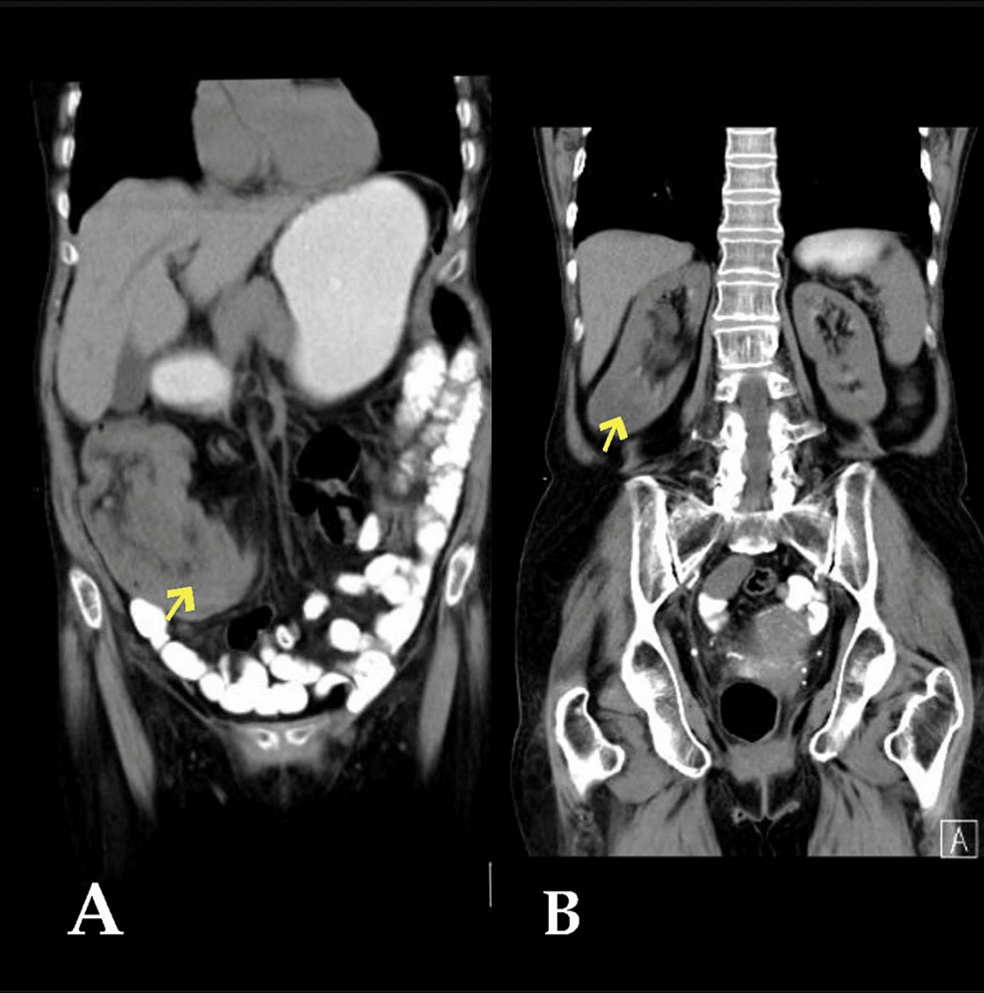
Case 2 - pre- and post-contrast CT scan of the abdomen. The images show (A) ileocecal intussusception reaching the mid-transverse colon with a suspected lesion at the apex of the invaginate bowel and (B) a rounded exophytic mass lesion from the lower pole of the right kidney.

In October 2020, an 86-year-old female medically free presented to the emergency department (ED) with four months history of intermittent abdominal pain in the right lower quadrant, aggravated by food, associated with nausea, anorexia, and weight loss. There was no history of vomiting, constipation, diarrhea, bloody stool, or fever. A thorough physical examination revealed mild tenderness over the right lower quadrant palpable mass. The patient’s workup showed a low level of hemoglobin (91 g/L), low MCV (71.4 fL), and low MCH (23.3 pg), which indicate microcytic hypochromic anemia. The computed tomography (CT) of the abdomen showed ileocecal intussusception reaching the mid-transverse colon with a suspected lesion at the apex of the invaginate bowel (Figures 3A, 3B). At the same time, in the CT, there was a rounded exophytic mass lesion from the lower pole of the right kidney. The patient underwent right hemicolectomy with right partial nephrectomy in the same session. Histopathological examination of the resected right colon showed a 6.5 cm mass in the cecum and ascending colon. Poorly differentiated adenocarcinoma (grade 3) with focal mucinous differentiation. The tumor invades through muscularis propria into pericolic tissue (PT3), 17 regional lymph nodes were free of tumor (PN0), and there were no microscopical evident malignant cells in surgical resection margins. The appendix was involved. And histopathological examination of the resected part of the right kidney showed <1 cm mass in the lower pole. The specimen was diagnosed as papillary renal cell carcinoma and oncocytoma. Post-operatively, the post-surgery tumor board decided for only follow-up and no more adjuvant therapy due to the performance status of the patient.

Case 3

A 56-year-old female patient known to have diabetes mellites (DM) presented to the emergency department complaining of abdominal pain for the last six months. She described her pain as colic with intermittent frequency and progressive over time. She didn’t notice any special aggravating or relieving factors. Her pain is associated with alternating bowel habits and attacks of bleeding per rectum. She has anorexia and has lost more than 10 kg. On examination, her abdomen was soft and non-tender with no palpable mass or organomegaly. Her digital rectal examination showed a mass, 4 cm from anal verge, which was nodular and easy to bleed. Hematological investigation results are as follows: WBC 6.9 x10^-9^/L, Hb 12.8 g/L, platelet count (PLT) 178x10^-9^/L, creatinine 105 umol/L, urea 6.5 mmol/L. No tumor markers were present. Her CT scan revealed a short segment of circumferential mural thickening of rectum surrounded by mild fat stranding. Left pelvic kidney with lower pelvicalyceal urothelium thickening and enhancement with speckles of calcifications. MRI of pelvis showed mid-rectal short segment of circumferential mural thickening about 6 cm from the anorectal angle surrounded with fat stranding and ill-defined enhancing rim of about 7 mm thickness into mesorectal fat. No clear lymph node involvement. Left side ectopic pelvic kidney with lower pole soft tissue enhancing mass lesion seen casting the lower calyx with heterogenous dominantly dark signal in T2 weighted image (T2WI) and her colonoscopy shows rectal mass, and endoscopic biopsy proves moderately differentiated adenocarcinoma. The patient was referred to urology after the CT findings, cystoscopy was done for her, and left renal biopsy was taken. The result of the biopsy was inconclusive. The case was discussed in the tumor board and decision was made for upfront low anterior resection and left nephrectomy. The patient underwent ultra-low anterior resection with coloanal anastomosis with covering loop ileostomy and left nephroureterectomy. The left kidney tumor was 4 cm and unifocal. Histopathology showed a non-invasive papillary urothelial carcinoma of renal pelvis, low grade (G1) pT1a. Margins were uninvolved and there was no lymphovascular invasion. No lymph nodes were submitted or found. According to pathologic stage classification (American Joint Committee on Cancer {AJCC}) it was pTa1Nx. The size of the rectal tumor was 4 cm and entirely below the anterior peritoneal reflection. Histopathology showed an invasive adenocarcinoma, low grade (G1) pT2N1a. Tumor invaded the muscularis propria. Margins were uninvolved and there was no lymphovascular or perineural invasion. Out of the 11 lymph nodes examined, only one lymph node was involved. According to pathologic stage classification (American Joint Committee on Cancer {AJCC}) it was pT2N1aMx. The case was discussed again post-operatively in tumor board and decision was made for adjuvant chemoradiation.

## Discussion

The incidence of (MPMTs) varies dramatically between antemortem and postmortem examinations, becoming a serious medical issue. Evidence shows that the overall incidence of MPMTs is between 2.4% and 17% [5-7]. As a result of the low prevalence of MPMTs and the wide range of clinical features that they might exhibit, most clinicians are unprepared to deal with their diagnosis and treatment. This can happen when clinicians misdiagnose MPMTs as a recurrence or metastasis of initial cancer that might lead to the use of ineffective treatment strategies. The propensity of certain individuals to acquire MPMTs (synchronous or metachronous) can be explained by an inherited predisposition or by the activity of carcinogenic factors on various organs at particular time periods. This is the most presumable explanation for the possible associations between slow-growing and aggressive tumors in our three cases. Multiple and single tumors have the same pathogenesis mechanisms, where a combined influence of environment and genetic factors promotes a new tumor's development. Thus, it could be assumed that the emergence of metachronous tumors is most likely the result of a combination of multifactorial and predisposing factors. However, it is harder to understand the development of synchronous tumors even if multifactorial and predisposing factors couldn't be ruled out, and their development appears to be more time-dependent [8]. All three cases we reported developed two histologically distinct malignancies, meeting the inclusion criteria of Warren and Gates to be defined as MPMTs [9].

In case 1, the immunohistochemical analyses on the two surgical specimens showed a moderately differentiated adenocarcinoma infiltrating through the muscularis propria into the tissues surrounding the colon and an invasive ductal carcinoma of the breast, which suggests this as a synchronous occurrence of tumors. The reported overall incidence of DPM has ranged from 3% to 20% among colorectal cancer patients; this frequency is comparatively higher among the general population [3]. The incidence of synchronous breast and colon cancers in women is reported to be 3.85% [10]. Uncertainty persists over the association between synchronous breast and colon cancers, which have not been thoroughly studied [11]. Synchronous tumors have a high disposition for family history. In individuals with hereditary breast and colorectal cancers, genetic mutation of CHEK2*1100delC (CHEK2) has been observed [12,13]. An increased risk of breast cancer by three to five times has been attributed to this low-penetrance breast cancer-predisposing gene [14]. Numerous epidemiological studies have examined the relationship between breast and colorectal cancer, examining the risk of developing a second primary non-colorectal cancer following colorectal adenocarcinoma, with the majority of those secondary malignancies occurring within three years of the primary colorectal cancer [15-18]. According to Ueno et al., when colon cancer was correlated with the existence of other tumors in women, the most commonly seen tumor was gastric cancer, followed by breast cancer [17]. The outcome of synchronous cancers relies on each cancer stage individually. Compared with single cancer, synchronous cancers have no worse prognosis with appropriate therapy. In our multidisciplinary team (MDT) cancer meeting, it was decided to do an open extended left hemicolectomy with primary colo-colonic anastomosis followed by bilateral mastectomy and bilateral sentinel lymph node biopsies of both axillae. The patient had received four cycles of neoadjuvant chemotherapy, and post-operatively the patient received adjuvant chemotherapy, radiotherapy, and hormonal therapy. If both tumors demand adjuvant chemotherapy, it is critical to think about what chemotherapy regimen should be administered post-operatively. However, the challenge is to find an anticancer therapy approach that incorporates both cancer types without increasing toxicity or appropriate pharmacological reactions and without having a negative impact on the overall prognosis. In case 2, the patient has a poorly differentiated adenocarcinoma (grade 3) of the colon with focal mucinous differentiation and papillary renal cell carcinoma (RCC) and oncocytoma synchronously. The coexistence of RCC with other neoplasms has already been reported in the literature. According to data from the Norwegian Cancer Registry, 18% of 1425 RCC patients who were studied over the course of seven years had at least one additional primary tumor [19]. A retrospective investigation conducted at a Japanese university hospital discovered a synchronous malignant tumor in 5.9% of patients who underwent RCC resection, and the presence of other primary tumors at the time of nephrectomy was found to be an independent predictor of post-operative survival [20]. Czene and Hemminki's study demonstrates unequivocally that individuals with RCC face an increased risk of developing additional malignancies not only during the first year following the main diagnosis but also after > 10 years [21]. A rare case of coexistence of three neoplasms (colonic carcinoma, renal cell carcinoma, and gastrointestinal stromal tumor) in a single patient has been reported [22]. It is suggested that microsatellite instability testing may be used in all patients presenting with colorectal and urological cancers to detect a common genetic aberration between malignancies [23]. Both RCC and colon cancer can have a genetic susceptibility caused by mutations in the c-MET and c-KIT proto-oncogenes, where both proto-oncogenes encode receptor tyrosine kinases [24]. However, this does not prove an etiological link between the two tumor types. With careful patient selection, therapeutic goals like improving symptoms, quality of life, and palliation can be achieved [25,26]. However, the optimal management of such cases remains unclear. In our case, a right hemicolectomy with right partial nephrectomy in the same session was performed. The use of en bloc resection has been found to have a significant role in locally advanced disease, although there is a lack of evidence supporting radical resection. In case 3 of our report, the MPMTs were diagnosed as a non-invasive papillary urothelial carcinoma of the renal pelvis, low grade (G1) and rectal tumor invasive adenocarcinoma, and low grade (G1) with an invasion of muscularis propria. Urothelial cancer (UC) is the fourth most common malignancy. The upper urinary tract (renal pelvis and ureter) urothelial cancer is rare, being only 5-10% of all UC. Upper tract UC (UTUC) is three times more common in males than in females and it peaks at the age of 70-90 years. The development of UTUC is linked to both hereditary and environmental factors [27]. A strong association has been found between upper urinary tract urothelial cell carcinomas (UUT-UCC) and hereditary non-polyposis colorectal cancer (HNPCC), as HNPCC carriers have a 22-fold higher risk of developing UUT-UCC than the general population [28]. HNPCC, also known as Lynch syndrome (LS), is an autosomal dominant cancer syndrome that is caused by a mutation in one of the DNA mismatch repair (MMR) genes (MLH1, MSH2, MSH6, and PMS2) that causes microsatellite instability (MSI). Among HNPCC-associated cancers, the prevalence of UC is 5%, which makes it the third most common cancer after colon cancer (63%) and endometrial cancer (9%). HNPCC is the most common hereditary form of colon cancer, responsible for 3% of all new colon cancer diagnoses and it carries a 70-90% lifetime risk of colorectal cancer [29,30]. A study that aimed to determine the association between colorectal cancer and urologic cancers by quantifying colorectal cancer (CRC) risk after urologic cancer, as well as the risk for urologic cancer after CRC in patients without a known genetic syndrome, concluded that a history of renal pelvis and ureteral cancers, especially when diagnosed before the age of 60, increase the risk for subsequent CRC by 80% and 44%, respectively. Likewise, a previous CRC, particularly in cases with MPMT, increases the risk of developing renal pelvis cancer by 59% and ureteral cancer by 100%. These findings suggest a possible common pathogenetic mechanism between urologic cancers and CRC [31].

## Conclusions

Since MPMTs are rare and have a wide range of clinical features, they might be misdiagnosed as metastasis or a recurrence of initial cancer. To ensure early detection, clinicians must always consider the possibility of the presence or development of a second primary cancer not only when treating a “cancer patient” but also during long-term follow-up.
